# Motor Vehicle Driving During Pregnancy Does Not Influence Uterine Contractions

**DOI:** 10.1155/ogi/3096143

**Published:** 2025-12-08

**Authors:** Riko Araki, Masahito Hitosugi, Kentaro Takahashi

**Affiliations:** ^1^ Department of Legal Medicine, Shiga University of Medical Science, Otsu, Shiga, Japan, shiga-med.ac.jp; ^2^ Department of Obstetrics and Gynecology, Hino Memorial Hospital, Gamou, Shiga, Japan

**Keywords:** accidents, cardiotocography, fetus, pregnant woman, uterine contraction

## Abstract

**Background:**

No studies have effectively clarified the relationship between uterine contractions and the act of acceleration or driving behaviors in pregnant women.

**Aims:**

To confirm the effect of driving a motor vehicle on physiological changes in pregnant women, we examined uterine contractions while driving.

**Materials and Methods:**

Seventeen pregnant women with a gestational age of 30–35 weeks were enrolled in this study. Uterine contractions were monitored remotely using a mobile delivery monitoring device. Triaxial acceleration of the vehicle, vehicle velocity, and vehicle kinematics were monitored using a driving recorder.

**Results:**

The average number of uterine contractions per 30 min in all participants was 1.0 (range, 0.26–2.0). When comparing the median vehicle velocity during a uterine contraction with that when there were no contractions, no significant difference was found (23 km/hour vs. 31 km/hour, *p* = 0.36). The prevalence of low velocity (20 km/hour or less) was significantly higher, and that of higher velocity (50 km/hour or more) was lower during a uterine contraction than with no contractions (*p* = 0.023 and 0.012, respectively). When comparing resultant vehicle acceleration, no significant differences were found between women with uterine contractions and those with no contractions. The distributions were similar before and immediately before a uterine contraction and with no contractions.

**Conclusions:**

Driving a motor vehicle should be considered a normal activity of daily life in pregnant women and seems unlikely to predispose to preterm birth.

## 1. Introduction

Worldwide, approximately 1.19 million people die as a result of road traffic crashes. Road traffic injuries are the leading cause of death among children and young adults aged 5–29 years, including pregnant women [1]. Motor vehicle collisions (MVCs) are also a leading cause of fetal death related to maternal trauma. Because these maternal or fetal deaths are preventable with timely management, greater focus on MVCs among pregnant women drivers is needed. Recently, issues related to gender equality have progressed dramatically worldwide. According to a study in a rural area of Japan, approximately 90% of women continue to drive a motor vehicle during their pregnancy [2]. However, because the abdomen of a pregnant woman protrudes to a varying degree depending on the gestational age, some pregnant vehicle drivers have concerns about whether they should continue driving. According to a study using the National Automotive Sampling System/Crashworthiness Database, abdominal protrusion in pregnant women did not affect the occurrence or severity of abdominal injuries in relatively low‐speed frontal collisions [3]. Therefore, safety among pregnant women drivers must be enhanced.

Several physical or psychological changes are considered to interact with driving behaviors among pregnant women. A population‐based, self‐matched, longitudinal cohort analysis of MVCs involving pregnant women drivers in Ontario, Canada, suggested that the risk of a serious MVC is significantly increased during the early second trimester of pregnancy [4]. In a multicenter cross‐sectional survey in Japan, tension and cramps in the lower abdomen, distraction, and irritability were reported to occur often during pregnancy and were independent contributory factors to MVCs or near‐miss incidents [5].

Arguments that motor vehicle driving influences the physiological states of both pregnant women and their fetus have been reported. Nakajima et al. examined the fetal heart rate and uterine contractions of healthy pregnant drivers with a gestational age of 28–37 weeks using a portable cardiotocogram (CTG) [6]. The authors suggested that the number of uterine contractions decreased significantly while driving (from 2.0 every 20 min before driving to 1.4 every 20 min while driving). In contrast, another study among pregnant women with a gestational age of 29–41 weeks suggested that the number of uterine contractions increased while driving a vehicle (from 0.62 every 30 min before driving to 4.9 every 30 min while driving) [7]. In these studies, the CTG used was not a mobile type, so the examiners rode in the vehicle with study participants and monitored them directly, possibly modifying the experimental circumstances while participants were driving. Also, drivers are engaged in multidirectional acceleration as well as behaviors such as pedaling, steering, or operating other equipment while driving. Therefore, when investigating the effects of motor vehicle driving on physiological changes in pregnant women, the effect of acceleration and drivers’ behaviors must be examined. However, the relationship between uterine contractions and the act of acceleration or driving behaviors in pregnant woman has remained unclear. Solving this issue may enhance the social participation of pregnant women in the form of vehicle driving and provide evidence for obstetricians and gynecologists to give appropriate advice to pregnant women regarding motor vehicle driving. To assess the effect of motor vehicle driving on physiological changes in pregnant women, in this study, we examined uterine contractions while driving among pregnant participants.

## 2. Materials and Methods

### 2.1. Participants

Seventeen pregnant women with a gestational age of 30–35 weeks were enrolled in this study. All had healthy singleton pregnancies and no previous diseases. Nine women were primipara, and eight were multipara, and none had a history of preterm labor, preterm delivery, or a history of cervical incompetence. All participants reported that they routinely drove a vehicle. The principle and procedure of this study were fully explained to all participants, and written consent was obtained. The Ethics Committee of Shiga University of Medical Science approved this study (no. R2020‐148).

### 2.2. Procedure

Uterine contractions were monitored remotely using a mobile delivery monitoring device (iCTG; Melody International, Kagawa, Japan). Participants could drive their own motor vehicle freely at any time, after attaching the monitoring device. We were able to remotely observe the CTG while the participant was driving. The time course of uterine pressure was observed by two certified midwives on the CTG. We also installed a driving recorder (DRV‐830; Kenwood, Yokohama, Japan) on the windshield of each participant’s vehicle to monitor triaxial acceleration of the vehicle, vehicle velocity, and vehicle kinematics. Triaxial acceleration is acceleration in longitudinal, lateral, and vertical directions. For pregnant women sitting correctly in the driver’s seat, the obtained acceleration was considered to be acting on the body and, therefore, recognized as applied acceleration or the act of acceleration. Vehicle kinematics means the movements of the vehicle such as going forward, turning right or left, or stopping.

### 2.3. Data Analysis

Uterine contractions were assessed by two certified midwives using the CTG. The conditions and vehicle kinematics during uterine contractions were monitored using the driving recorder. Furthermore, to obtain similar information at the time without uterine contractions, driver kinematics as well as vehicle acceleration and velocity were obtained every 2 min before and after a uterine contraction. One pregnant woman who did not have any uterine contractions while driving was excluded from the subsequent analyses.

Using the obtained data on triaxial vehicle acceleration, the resultant acceleration applied to the body was calculated as the square root of the sum of the square of each axis acceleration.

### 2.4. Statistical Analysis

Continuous variables are shown as mean ± standard deviation for values that followed a normal distribution, and as median and interquartile range for values that were not normally distributed. Chi‐square tests were used to compare the prevalence between two groups. The Student’s *t*‐test was used to compare the values between two groups with a normal distribution. A Mann–Whitney test was conducted for values without a normal distribution to identify differences in values between two groups. The analysis was performed using statcel4 (OMS Publishing, Tokyo, Japan). A *p* value of less than 0.05 was considered statistically significant.

## 3. Results

**Figure 1:**
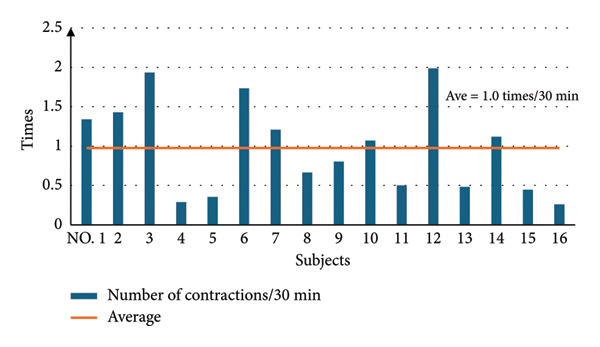
Number of uterine contractions over 30 min while driving. Bold horizontal line is average number of uterine contractions in all participants (1.0/30 min).

Seventeen pregnant women were enrolled in this study, with a mean age of 31.3 ± 3.3 years and mean gestational age of 32.6 ± 1.8 weeks. The prevalence of uterine contractions per 30 min in each pregnant woman is shown in Figure 1. The average number of uterine contractions in all participants was 1.0 ± 0.6, with a range from 0.26 to 2.0 contractions. No participants perceived painful uterine contractions that disturbed their driving.

### 3.1. Vehicle Velocity and Uterine Contraction

**Figure 2:**
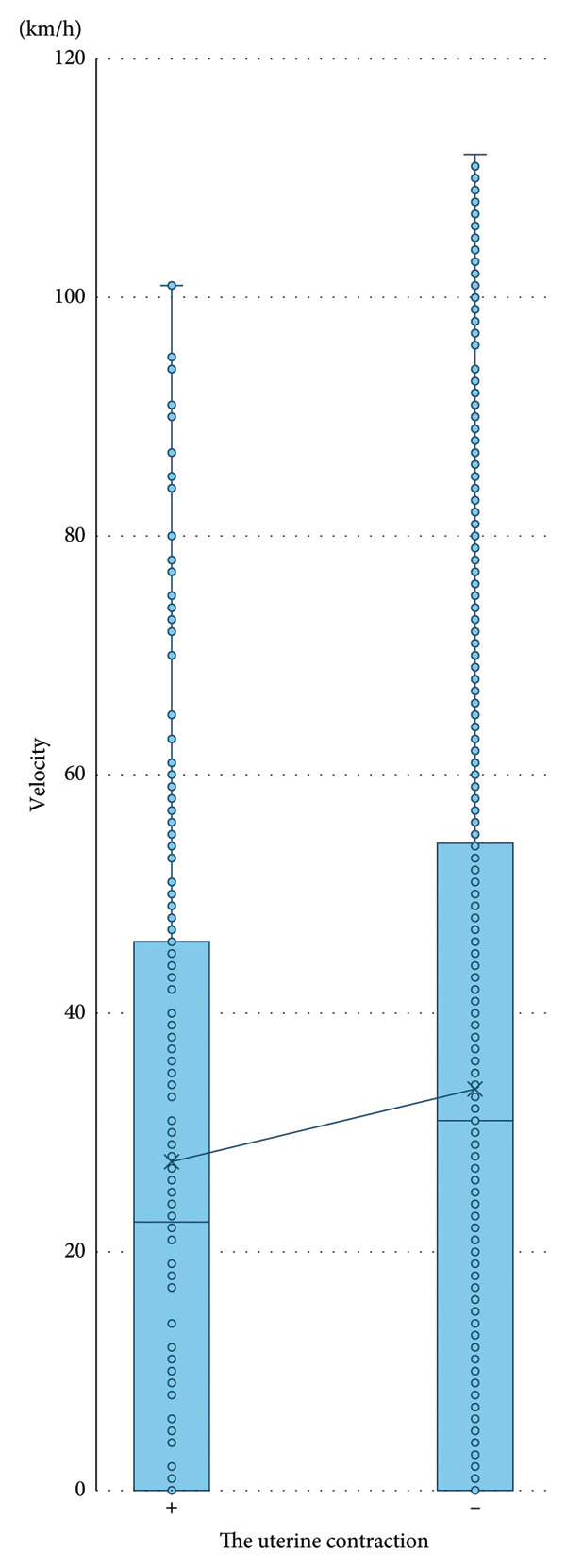
Comparison of vehicle velocity during uterine contractions and with no contractions.

**Figure 3:**
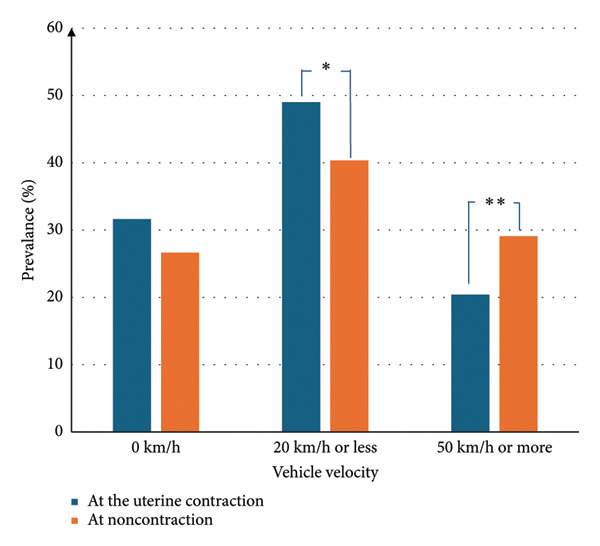
Comparison of vehicle velocity during uterine contractions and with no contractions. Significant differences were found between a vehicle velocity of 20 km/hour or less and 50 km/hour or more (^∗^ *p* = 0.023 and ^∗∗^ *p* = 0.012, respectively).

We examined the relationship between vehicle velocity and uterine contractions. No significant difference was found in the median vehicle velocity when there were uterine contractions compared with when there were no contractions (median velocity 23 km/hour vs. 31 km/hour, *p* = 0.36) (Figure 2). We further compared the distribution of driving velocity during uterine contractions and with no contractions (Figure 3). The prevalence of low velocity (20 km/hour or less) was significantly higher during uterine contractions than with no contractions (*p* = 0.023). Furthermore, the prevalence of higher velocity (50 km/hour or more) was lower during uterine contractions than with no contractions (*p* = 0.012).

### 3.2. Applied Acceleration and Uterine Contraction

We examined the relationship between applied resultant acceleration and uterine contractions in pregnant women. No significant association with vehicle acceleration was found between having uterine contractions and not having contractions (median 9.8 m/s^2^ vs. 9.8 m/s^2^, *p* = 0.41).

### 3.3. Vehicle Kinematics and Uterine Contraction

**Figure 4:**
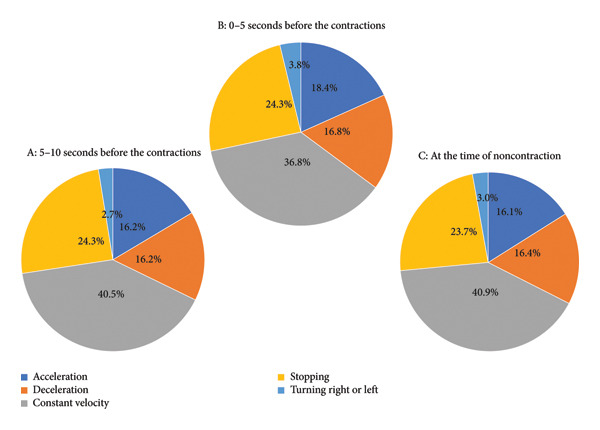
Distribution of vehicle movements before and immediately before uterine contractions, as well as with no contractions ((a) 5–10 s before contraction, (b) 0–5 s before contraction, and (c) no contractions).

We examined the effect of vehicle kinematics on uterine contractions. We examined the kinematics of the vehicle before (5–10 s prior) and immediately before (0–5 s prior) a uterine contraction. The distributions were compared during three time periods (5–10 s before a contraction, Figure 4(a); 0–5 s before a contraction, Figure 4(b); with no contractions, Figure 4(c)). The distributions were similar before and immediately before uterine contractions, and with no contractions, under the following conditions: moving at a constant velocity in 36.8%–40.9% of cases; stopping in 23.7%–24.3%; during deceleration in 16.2%–16.8%; during acceleration in 16.1%–18.4%; and turning in 2.7%–3.8% of cases.

## 4. Discussion

In this study, we first investigated the effects of vehicle velocity, acceleration, and kinematics on uterine contractions in pregnant women. The results suggested that the average number of uterine contractions while driving a motor vehicle was 1.0 every 30 min, with a range from 0.26 to 2.0. This result is similar to the number of uterine contractions at rest in late‐term pregnancy [8]. The frequency of uterine contractions has been examined in various lifestyle situations. In a study among 81 low‐risk pregnant women, physical activities such as prolonged standing, heavy housework, lifting, or organized exercise did not affect the rate of uterine contractions [9]. That study also suggested that climbing stairs and walking were associated with increased contraction frequencies [9]. One report found that a short‐term submaximal bicycle ergometer test induced uterine contractions in late‐term pregnant women with hypertension but not in healthy pregnant women [10]. When pregnant women at term performed exercise at a heart rate of 140 beats/minute on a cycle ergometer, uterine activity increased significantly during the exercise period, with a 5.5‐fold increase in contraction frequency [11]. These findings show that with a physical load, uterine contractility increases with moderately or more strenuous maternal exercise owing to the activation of the sympathetic nervous system. According to our results, motor vehicle driving does not affect uterine contractions because sitting in a motor vehicle and steering or pedaling does not result in a large physical load. Our results therefore suggest that driving a vehicle is unlikely to predispose to preterm birth.

Our analysis showed that among pregnant women in our study, the uterus contracted more often at low vehicle velocity (20 km/hour or less) than at a higher velocity (50 km/hour or more). There have previously been inconsistent theories regarding the effect of motor vehicle driving on uterine contractions. Our study was in accordance with a previous report that uterine contractions more often occur while the vehicle is stopped or immediately after the vehicle begins moving [7]; however, detailed data were unavailable in that study. In our study, pregnant women drove a vehicle daily in the vicinity of their home and workplace. The category of vehicle velocity of 20 km/h or less included situations in which the vehicle may be stopped or beginning to move. In these situations, drivers usually must pay greater attention and concentrate more than when moving at relatively higher velocity. Therefore, when comparing driving conditions, we found that uterine contractions occurred more often under conditions when the driver may be more tense or alert, such as when slowing or stopping for pedestrians. We hypothesize that, in these situations, the sympathetic nervous system is activated owing to a mental load. To confirm this hypothesis, further studies are required that measure pregnant women’s sympathetic response during vehicle driving. However, the findings of this study are novel and contribute new insights into the effects of driving in pregnant women.

Our results revealed the effects of acceleration or vehicle kinematics on uterine contractions. In this study, we examined vehicle acceleration via three aspects. While driving, the driver is affected in an anterior–posterior direction when accelerating, in lateral directions when the vehicle turns or swerves, and in a vertical direction when the vertical goes over a bump. There are no studies suggesting thresholds with respect to acceleration for pregnant women. Therefore, as we calculated the resultant acceleration, we were able to determine the acceleration actually applied to the body. In this study, we first assessed the acceleration applied by pregnant women vehicle drivers. No significant differences were found in the acceleration during a uterine contraction compared with when there were no contractions. Their median values were 9.8 m/s^2^ in each, acceleration equivalent to 1 × gravity. Our findings suggest that a vehicle acceleration of approximately 1 × gravity is safe for pregnant women.

In comparing the effect of vehicle kinematics on pregnant women before or immediately before a uterine contraction and with no contractions, we found no marked differences. This suggests that the requirements of daily motor vehicle driving, such as recognition and alertness, decision‐making, steering, and pedaling, do not influence contraction of the uterus in pregnant women.

This study has some limitations. First, the sample size was relatively small. This is because we required the women in the study to attach a mobile delivery monitoring device in a suitable position before vehicle driving. This was quite complex and made it difficult to recruit many participants. However, we believe that our results are reliable enough to provide useful information. Second, we monitored different vehicle situations using a driving recorder; however, we did not analyze details of all circumstances, such as the time (night or day) or weather (sunny or rainy). In the future, the specific circumstances while driving a vehicle should be considered in the analysis. Third, no study participants experienced an MVC or near‐miss incident while driving. Long‐term monitoring may better capture the effects of such high‐risk conditions. Further studies would clarify the effect on uterine contractions of collisions or near‐miss situations while driving. Fourth, the women who participated in this study all had singleton pregnancies without complications. It is possible that pregnant women experiencing complications would be more susceptible to the acceleration levels in this study. Additionally, one in every 50 births in Japan is currently a multiple birth [12]. There are some physiological and morphological differences between singleton and multiple pregnancies, and our results may therefore not hold for women with multiple pregnancies. Further studies among pregnant women with complications or with multiple pregnancies are required to confirm our results.

## Conflicts of Interest

The authors declare no conflicts of interest.

## Funding

The authors received no specific funding for this work.

## Data Availability

The data that support the findings of this study are available on request from the corresponding author. The data are not publicly available due to privacy or ethical restrictions.
